# Mechanical Response Changes in Porcine Tricuspid Valve Anterior Leaflet Under Osmotic-Induced Swelling

**DOI:** 10.3390/bioengineering6030070

**Published:** 2019-08-15

**Authors:** Samuel D. Salinas, Margaret M. Clark, Rouzbeh Amini

**Affiliations:** Department of Biomedical Engineering, The University of Akron, Akron, OH 44325, USA

**Keywords:** biaxial mechanical testing, cardiac valves, osmotic swelling

## Abstract

Since many soft tissues function in an isotonic in-vivo environment, it is expected that physiological osmolarity will be maintained when conducting experiments on these tissues ex-vivo. In this study, we aimed to examine how not adhering to such a practice may alter the mechanical response of the tricuspid valve (TV) anterior leaflet. Tissue specimens were immersed in deionized (DI) water prior to quantification of the stress–strain responses using an in-plane biaxial mechanical testing device. Following a two-hour immersion in DI water, the tissue thickness increased an average of 107.3% in the DI water group compared to only 6.8% in the control group, in which the tissue samples were submerged in an isotonic phosphate buffered saline solution for the same period of time. Tissue strains evaluated at 85 kPa revealed a significant reduction in the radial direction, from 34.8% to 20%, following immersion in DI water. However, no significant change was observed in the control group. Our study demonstrated the impact of a hypo-osmotic environment on the mechanical response of TV anterior leaflet. The imbalance in ions leads to water absorption in the valvular tissue that can alter its mechanical response. As such, in ex-vivo experiments for which the native mechanical response of the valves is important, using an isotonic buffer solution is essential.

## 1. Introduction

The properties of many soft tissues can best be obtained by conducting experiments in an environment that resembles in-vivo conditions. In the realm of biomechanics, the characterization of soft tissue mechanical properties has traditionally relied on benchtop experiments such as uniaxial or biaxial tensile extension tests [1,2]. For these experiments, both for the purpose of tissue storage and during the course of the experiments, tissue samples are generally immersed in a buffer solution (e.g., phosphate buffered saline (PBS)). By virtue of its non-toxicity to cells and its pH buffering capability, PBS is widely used in biological studies [3]. 

The regulation of osmolarity is also of great importance in maintaining cell and tissue viability. Researchers in previous studies have quantified the effects of hypo- and hyper-osmolarity on soft tissues [4,5,6,7,8]. The low concentration of ions in a hypo-osmotic solution ultimately leads to tissue swelling over time. The subsequent changes in morphology of the tissue and the potential for damage to its constituents could lead to alteration of the mechanical responses [9,10]. Moreover, Lanir et al. have shown the effects of swelling and their correlation with residual stresses, as demonstrated in the left ventricle and aortic tissues of murine models [5,10,11]. Despite such strong evidence of mechanical dependence on normal osmolarity, deionized (DI) water has been employed in some studies in lieu of isotonic solutions [12,13]. In one particular in-vitro study conducted on heart valves, the assumption of no potential difference between DI water and PBS as it pertains to the mechanical responses of the tissues was adopted [12]. Notwithstanding the importance of the findings of these studies, isotonic solutions have been generally used in similar ex-vivo valvular studies to prevent changes in the mechanical responses of heart valves [14,15,16,17]. Since no previous experiments have been conducted to specifically show the effects of hypotonicity on the mechanical response of cardiac valves, we performed experiments on porcine tricuspid valve (TV) anterior leaflets in order to guide future research in heart valve biomechanics. 

The TV, which is located on the pulmonary side of the heart, is composed of three leaflets: anterior, posterior and septal leaflets. The study of the biomechanics of this valve, albeit nascent in comparison to the study of mitral valve biomechanics, has seen an emergence in interest [16,18,19,20,21,22,23,24,25,26]. The TV is characterized by having a larger orifice than the mitral valve as well as having thinner leaflets [27]. The extracellular matrix (ECM) in both heart valves is comprised primarily of collagen, elastin, and proteoglycans; and hence, it plays a role in the mechanical response of the valve tissue [28,29,30]. In this brief study, we hypothesize that the swelling effect due to the exposure of TV anterior leaflets to DI water will alter their mechanical response.

## 2. Materials and Methods 

### 2.1. Specimen Preparation

Porcine hearts (n=14) were acquired from a local slaughterhouse (3-D Meats, Dalton, OH, USA) and transported in chilled PBS back to our laboratory. Consistent with our previous methodology [31], upon isolating the TV apparatus, we identified and excised the anterior leaflet. The tissue was later trimmed to a smaller square size (approximately 11 mm × 11 mm) using a custom-made tissue phantom while ensuring that the axes of the tissue phantom coincided with the radial and circumferential anatomical directions of the tissue samples as described previously [31]. The radial direction was defined perpendicular to the TV annulus and the circumferential direction was defined as the direction perpendicular to the aforementioned radial direction [32].

Prior to mounting the anterior leaflet specimen on a custom-built biaxial tensile machine [31,33], the thickness of each specimen was measured using a thickness gauge. Five readings were taken from each sample with the average value used in our calculations. Next, four glass submillimeter markers were attached on the surface of the leaflet for optical tracking of tissue deformation. Suture lines were attached around the edges of the tissue. The dimensions of the trimmed tissue enclosed by the suture lines were 7.6 mm × 7.6 mm.

### 2.2. Biaxial Testing Protocol

The maximum right ventricular pressure for a normal person is defined as 30 mmHg [34,35]. From our previous study of the porcine TV, we found that the average thickness of the anterior leaflet was 313 μm [31]. In our prior work, we also employed Laplace’s law to arrive at an estimated stress value for the leaflets. Given the above parameters, the maximum target stress used in this study was calculated to be 127 kPa. A total of five loading protocols, listed in Table 1, were employed. 

Each protocol consisted of ten loading/unloading cycles. Only data from the tenth cycle was used in our analysis; the first nine were used for pre-conditioning purposes. The bath was filled with room temperature (21 °C) PBS and the specimen was loaded on the biaxial actuators. A tare load of 0.5 grams was used throughout the biaxial testing of the anterior leaflets. It is important to note that the load applied to achieve the desired stress was dependent upon the sample thickness. Hence, each sample had unique loads applied to it.

### 2.3. Tissue Swelling Application

Following all five loading protocols, tissue samples were unmounted and placed in DI water. In previous in-house tests (data not shown), we had observed that the maximum swelling of porcine TV leaflets occurred after they were immersed in DI water for two hours. As such, we used a two-hour submergence period for all samples in this study. Following the two-hour soaking period, the specimen thickness was measured again. Before being remounted on the biaxial testing machine, the samples were retrimmed to the 11 mm × 11 mm specimen size mentioned above. All protocols from Table 1 were then repeated. Similarly, our control group (n=14), which used a subset of specimens soaked in PBS for 2 hours, underwent the same testing procedure as specimens that were soaked in DI water. Because the control samples did not change in size, no specimen retrimming was necessary.

### 2.4. Data Processing

Data collected from biaxial testing was analyzed using an internally developed program in MATLAB (MathWorks, Nantick, MA, USA). Positional data obtained from tracking the surface-mounted glass fiducial markers allowed for the calculation of the deformation gradient tensor, F, as described previously [31,36].

The Green–Lagrangian strain tensor, E, was then calculated:

(1) $$E=\frac{1}{2}\left(F^{T}F-I\right)$$

where I is the identity matrix. 

The load applied on the specimen allowed for the calculation of the first Piola-Kirchoff stress:

(2) $$P_{rr}=\frac{F_{r}}{A},\;P_{cc}=\frac{F_{c}}{A}$$

where *F* is the force applied by the actuators and *A* is the cross-sectional area, which is defined as the product of the length (7.6 mm) and thickness of the sample. The double subscripts *rr* and *cc* designate the radial and circumferential normal stresses, respectively. Likewise, the single subscripts *r* and *c* refer to the applied force in the radial and circumferential directions, respectively.

### 2.5. Statistical Analysis

To determine the effect of soaking in DI water on the mechanical response of the leaflet, a Student’s paired *t*-test was used. The null hypothesis for this analysis was that the average strain at an equibiaxial load of 85 kPa following DI water exposure was equivalent to the average strain prior to DI water submersion. A value of *p* ≤ 0.05 was considered as significant for this test. Only the equibiaxial data were used in the current analysis; data from other protocols may be used for future analyses, if needed.

## 3. Results

**Figure 1:**
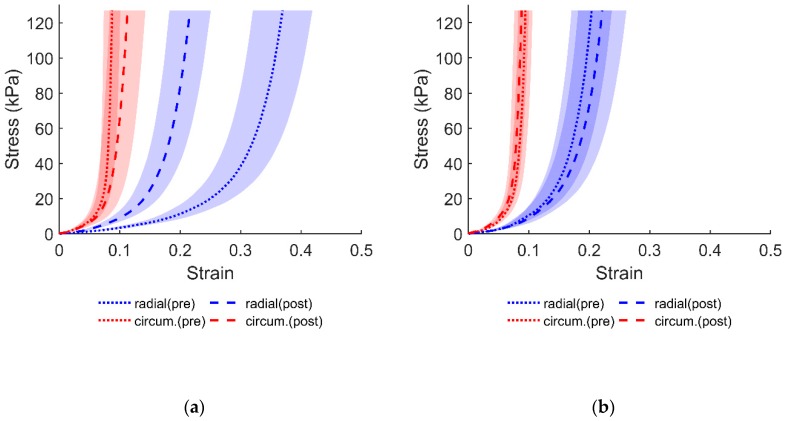
Average equibiaxial response of the anterior leaflet (i.e., Protocol 1 in Table 1) from: (**a**) DI water group; (**b**) control group (soaked in PBS). Shaded regions represent standard error.

Following two hours of soaking in DI water, the tissue exhibited less compliance in the radial direction as compared to the circumferential direction, as can be noticed from the results in Figure 1. Visual examination of the equibiaxial data (Figure 1) shows that the mechanical response in the control group exhibited a slight increase in radial strain. The additional protocols from Table 1 display similar behaviors, primarily concerning the radial compliance. Relative to the DI water group, the change in stress–strain response, albeit different, was not as aberrant in the control group. Subsequent protocols in the control group also showed a slight compliance in the radial direction similar to what is displayed in Figure 1. The stress–strain responses for the additional protocols, from Table 1, are provided in the Supplementary Materials.

The effect that DI water had on the leaflet shape and color was obvious upon visual inspection, since the tissue absorbed water during the soaking process. Following the two-hour soaking in DI water, the tissue thickness increased by 107.3% versus 6.8% in the control group that was soaked in PBS (Table 2). A Student’s paired *t*-test comparing pre- and post-treatment thickness across both DI and Control groups revealed that while both groups were significantly different, the p-value for the DI treatment group (10^−5^) was much smaller than that of the control (p=0.002).

**Figure 2:**
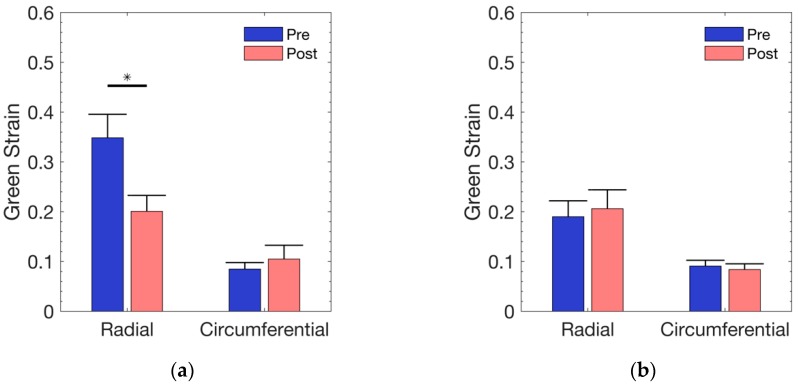
Average equibiaxial strain across all samples at an estimated physiological stress level of 85 kPa in: (**a**) DI water group; (**b**) control group. Error bars represent standard error.

At a mean normal ventricular pressure of 25 mmHg [37], the physiological strain using Laplace’s law (as mentioned above) was approximated to be 85 kPa. As shown in Figure 2, the treatment group exhibited a significant (*p =* 0.0026) radial reduction (34.8% to 20%) following exposure to DI water. The change in circumferential strain was neither visually detectable (8.4% to 10.4%) nor statistically different (*p =* 0.5176). Although the control group, in Figure 2, showed a minimal change in radial strain (19% to 20.6%) and circumferential strain (9% to 8.3%), the strains were not found to be significantly different in either the radial (*p* = 0.426) or the circumferential (*p* = 0.546) direction.

## 4. Discussion

The aim of this study was to determine the validity of the use of DI water, in lieu of isotonic solutions, as a viable medium for the handling of heart valve tissues during experiments that rely on obtaining native mechanical responses of the tissue. PBS, as a commonly used isotonic solution, does present various benefits for experiments involving biological specimens. Primarily, the isotonic properties of PBS ensure cell and tissue viability by approximating a physiological environment that is appropriate for data collection in research. Our study has shown that for equibiaxial tests, the response in the radial direction is greatly affected by the hypo-osmotic environment introduced by DI water. Although the changes in the circumferential direction seem to be much smaller, due to the tensorial nature of strain, any changes in one component of the strain (in this case the normal strain in the radial direction) is indicative of a completely different state of deformation in the tissue. 

The stiffer tissue response in the radial direction (as shown in Figure 1) may be indicative of a restrictive ECM environment as a result of tissue swelling and the stretching of fibers [11]. The submersion of soft tissues in solutions with different osmotic conditions is known to affect tissue morphology by changing the intrafibrillar water [7]. This change was clearly seen in the TV samples following their submersion in DI water. In fact, not only was the specimen thickness affected (Table 2), but the overall dimensions of the tissue were also altered. Interestingly, statistical analysis performed on the thickness in both groups revealed that both were significantly different, albeit the DI treatment group had a much smaller *p*-value. While a significant thickness difference was not expected in the control group, such differences did not lead to significant changes in the mechanical responses of the tissue. 

It has been shown that ECM collagen fibers are generally undulated in the unloaded state and, with increases in deformation leading to uncrimping of the fibers, they become stiffer [38]. The TV anterior leaflet, however, has a complex microstructure that affects its tissue-level mechanical response [39]. As such, understanding the overall effects of swelling-induced uncrimping and their influence on the mechanical properties of the TV leaflets will require further investigation. 

In a previous study, Pierce et al. examined the effects of exposure to hypotonic solutions on the mechanical responses of the mitral valve annulus as a pilot study [12]. They used an indentation method and compared the measured forces for the same level of indentation depth in samples submerged in DI water as compared to those immersed in an isotonic saline solution. Even with a small sample size, the mean force recorded for the control group was smaller than that of the hypotonic group, indicating the potential stiffening of the tissue. Although no statistically significant difference was found, it is worth noting that a small sample size was used, and the standard deviations were relatively large. While caution should be taken in comparing biaxial testing and indentation and also in extending the findings for one type of cardiac valve to another, we believe that with an increased sample size, the investigations of Pierce et al. would likely corroborate the findings of our study.

Our study was not without limitations. Although the statistical analysis performed on the control group did not indicate a significant difference in response due to immersion in an isotonic PBS solution, one should recognize that time also plays a degenerative role in tissue integrity. Our current method was not able to isolate such potential effects on the tissue mechanical responses. It is possible that with longer immersion in an isotonic PBS solution at room temperature (21 °C), tissue degeneration leads to alteration of the mechanical responses of the tissue. In addition, the focus of this study was on the role of isotonicity on the biomechanical responses of the tricuspid valves, especially as it pertains to ex-vivo setups [14,15]. However, in functioning valves, the viscosity of the flow could significantly affect the flow profile and subsequently alter the deformation of the valve leaflets. As shown by Biswas et al., even viscosity-matched media may not biomechanically function in a manner similar to native blood, and such limitations should always be considered in the interpretation of the results of ex-vivo studies [40]. Another limiting factor in interpretation of our data is the existence of a significant difference between the radial responses at estimated physiological stress in the DI group prior to DI water exposure and in those of the control group (*p*-value = 0.0174, Student’s paired *t*-test). Since no known errors existed in our methodology, we attributed such differences to possible variations in the porcine hearts obtained from the local slaughterhouse. Despite such differences, in our opinion, the important outcome of this study, i.e., exposure to DI water significantly changes the mechanical responses of the tricuspid valve leaflets, is still valid as it relies on the statistical comparison within each of the two groups rather than a comparison between the two groups. Lastly, tissue swelling encompasses not only physical changes in specimen thickness but also volume. Since the biaxial tensile machine is able to track the fiducial marker configuration, any changes in tissue can be tracked. However, following DI water application and tissue swelling, the physical changes in the specimen required that the tissue be retrimmed to the 11 mm × 11 mm size discussed in Section 2.1. This measure required that a new set of markers be attached, thereby losing the original marker configuration that would allow for the quantification of the induced physical changes using the marker tracking tool in our biaxial testing device.

## 5. Conclusions

The effect of DI water on the anterior TV leaflet yielded a mechanical response that was significantly different from the response of valves that were immersed in an isotonic PBS solution. Such outcomes further support the importance of using an isotonic solution when conducting experiments that require mimicking the in-vivo mechanical response of cardiac valve tissues.

## Figures and Tables

**Table 1:** Loading protocol for radial and circumferential directions.

Protocol	Radial (kPa)	Circum. (kPa)
1	127	127
2	95.25	127
3	127	95.25
4	63.5	127
5	127	63.5

**Table 2:** Average thickness of anterior leaflets in DI water group and control group.

Heart	DI-Pre	DI-Post	Control-Pre	Control-Post
1	424	924	269	299
2	391	627	237	325
3	314	662	342	393
4	259	490	312	350
5	246	467	287	284
6	317	548	233	259
7	254	482	287	284
8	332	416	223	226
9	337	614	246	251
10	299	599	223	228
11	322	609	348	378
12	350	548	343	396
13	224	1061	335	365
14	315	1046	269	279
Average	313	650	289	308
Std. Dev.	53	201	44	57
*p*-value	<0.001		0.002	

Note: All thickness values reported in this table are in microns.

## Supplementary Materials

The following are available online at https://www.mdpi.com/2306-5354/6/3/70/s1, Figure S1: Protocol 2 for DI water group, Figure S2: Protocol 3 for DI water group, Figure S3: Protocol 4 for DI water group, Figure S4: Protocol 5 for DI water group, Figure S5: Protocol 2 for control group, Figure S6: Protocol 3 for control group, Figure S7: Protocol 4 for control group, Figure S8: Protocol 5 for control group.
