# Supplementary material for: Mechanical Response Changes in Porcine Tricuspid Valve Anterior Leaflet Under Osmotic-Induced Swelling
Source: Bioengineering (Basel). 2019 Aug 15;6(3):70. doi: 10.3390/bioengineering6030070 (PMC6784000; doi:10.3390/bioengineering6030070)
Supplement: Supplementary file 1 [file bioengineering-06-00070-s001.pdf]

bioengineering

Communication

Mechanical Response Changes in Porcine Tricuspid Valve Anterior Leaflet Under Osmotic-Induced Swelling

Samuel D. Salinas, Margaret M. Clark and Rouzbeh Amini *

[1] Department of Biomedical Engineering, The University of Akron, Akron, OH USA
* Correspondence: ramini@uakron.edu; Tel.: (330)-972-5232

Supplementary materials

Figure S1: Protocol 2 for DI water group

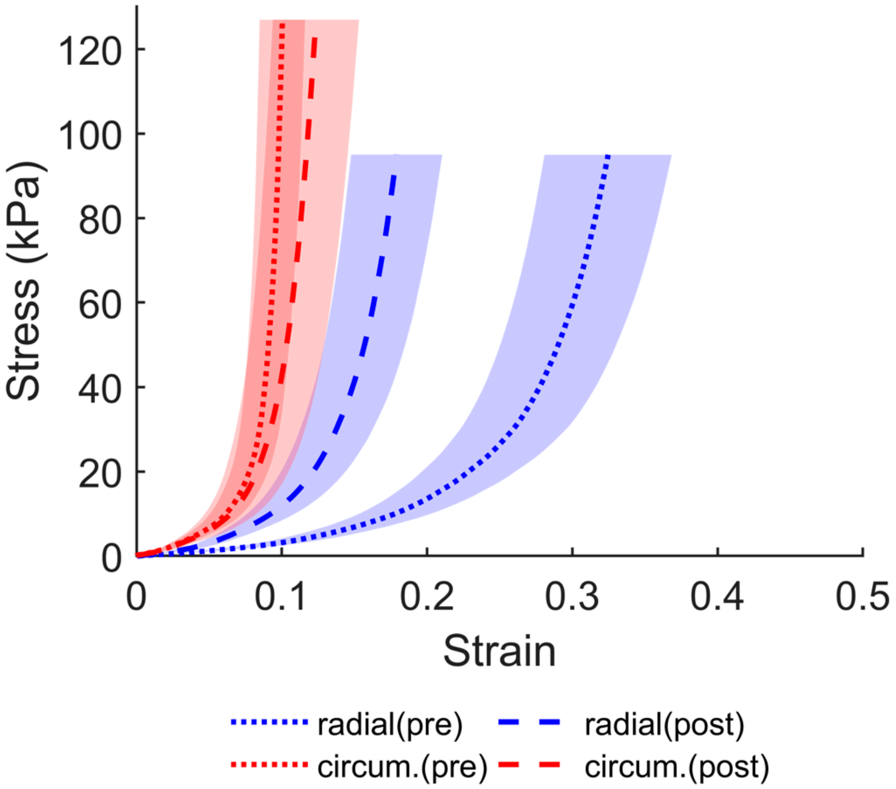

Figure S1. Average response of the anterior leaflet in the experiments with the maximum stress value of 95.25 kPa and 127 kPa in the radial and circumferential (circum.) directions, respectively (i.e., Protocol 2 in Table 1) for the DI water group. Shaded regions represent standard error.

Figure S2: Protocol 3 for DI water group

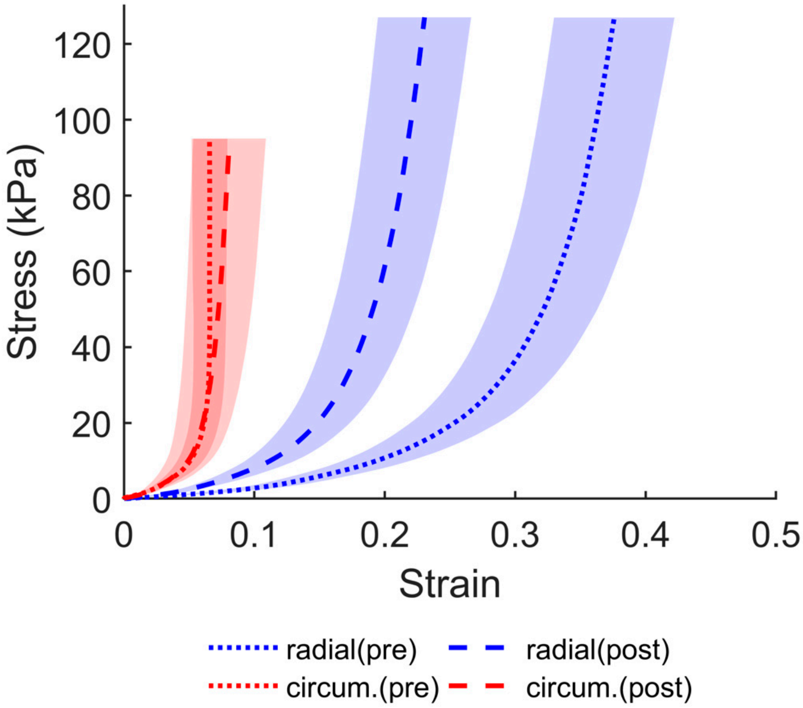

Figure S2. Average response of the anterior leaflet in the experiments with the maximum stress value of 127 kPa and 95.25 kPa in the radial and circumferential (circum.) directions, respectively (i.e., Protocol 3 in Table 1) for the DI water group. Shaded regions represent standard error.

Figure S3: Protocol 4 for DI water group

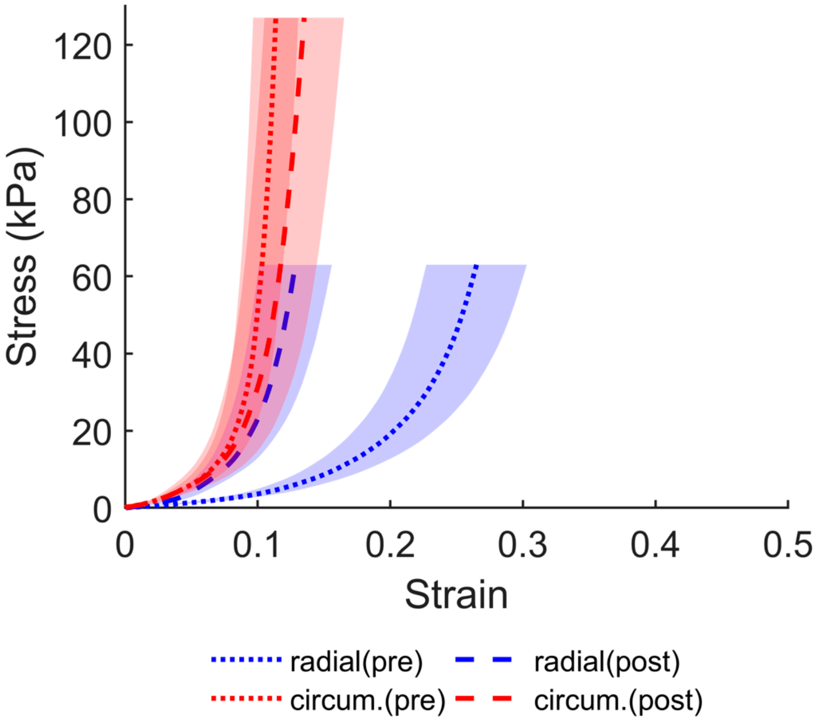

Figure S3. Average response of the anterior leaflet in the experiments with the maximum stress value of 63.5 kPa and 127 kPa in the radial and circumferential (circum.) directions, respectively (i.e., Protocol 4 in Table 1) for the DI water group. Shaded regions represent standard error.

Figure S4: Protocol 5 for DI water group

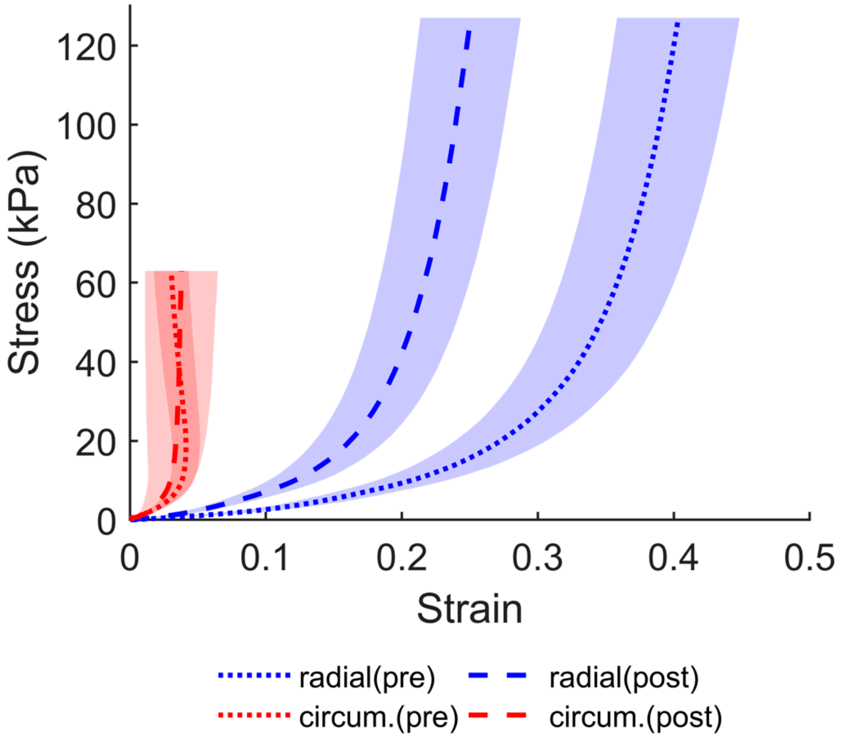

Figure S4. Average response of the anterior leaflet in the experiments with the maximum stress value of 127 kPa and 63.5 kPa in the radial and circumferential (circum.) directions, respectively (i.e., Protocol 5 in Table 1) for the DI water group. Shaded regions represent standard error.

Figure S5: Protocol 2 for control group

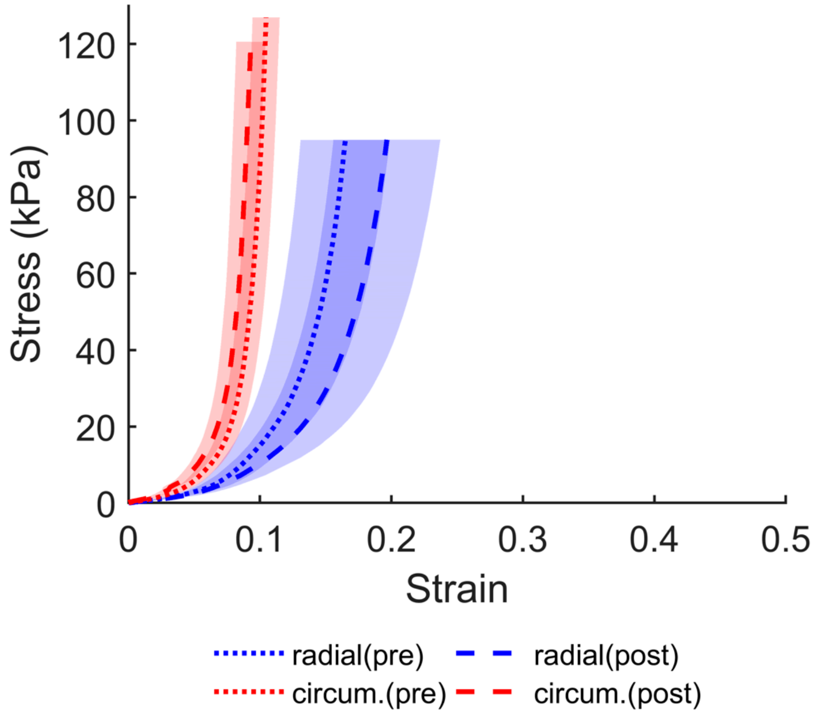

Figure S5. Average response of the anterior leaflet in the experiments with the maximum stress value of 95.25 kPa and 127 kPa in the radial and circumferential (circum.) directions, respectively (i.e., Protocol 2 in Table 1) for the control group. Shaded regions represent standard error.

Figure S6: Protocol 3 for control group

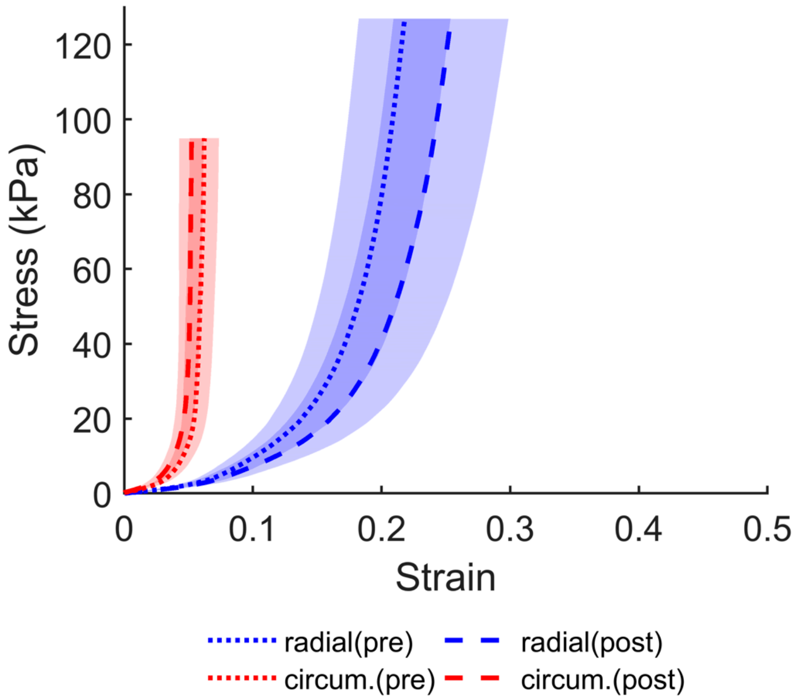

Figure S6. Average response of the anterior leaflet in the experiments with the maximum stress value of 127 kPa and 95.25 kPa in the radial and circumferential (circum.) directions, respectively (i.e., Protocol 3 in Table 1) for the control group. Shaded regions represent standard error.

Figure S7: Protocol 4 for control group

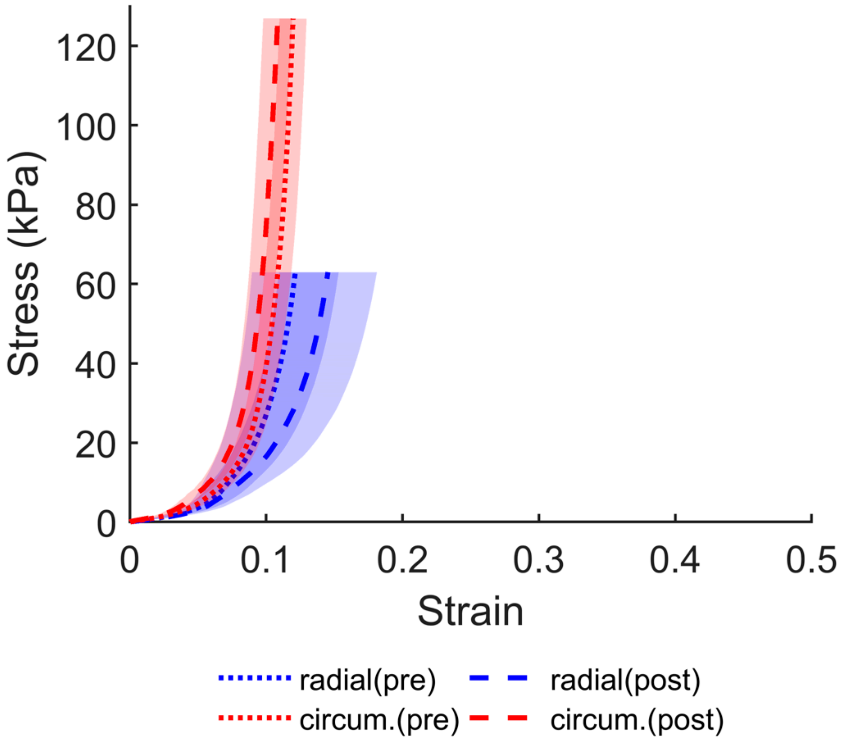

Figure S7. Average response of the anterior leaflet in the experiments with the maximum stress value of 63.5 kPa and 127 kPa in the radial and circumferential (circum.) directions, respectively (i.e., Protocol 4 in Table 1) for the control group. Shaded regions represent standard error.

Figure S8: Protocol 5 for control group

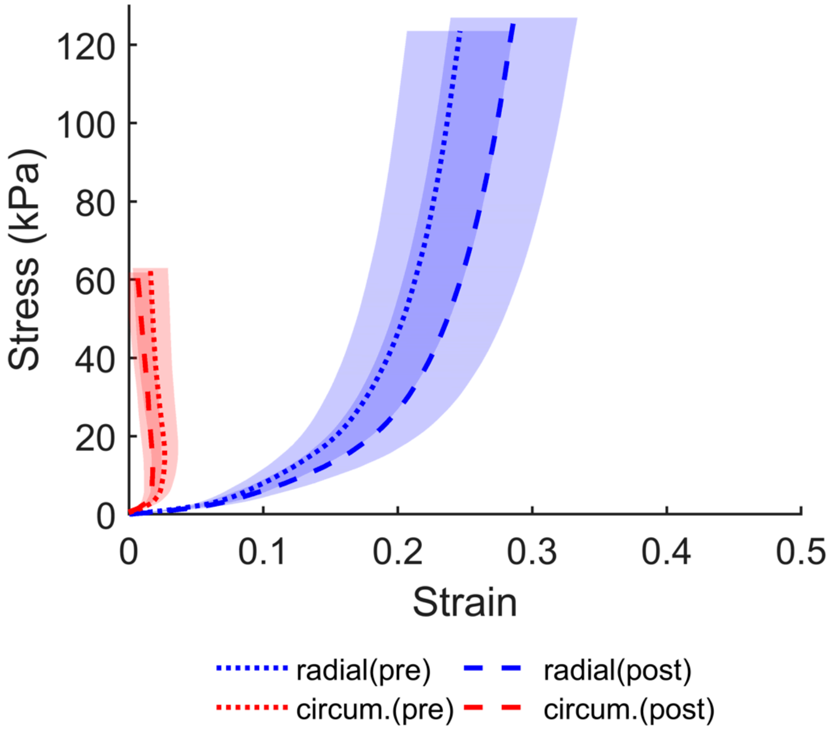

Figure S8. Average response of the anterior leaflet in the experiments with the maximum stress value of 127 kPa and 63.5 kPa in the radial and circumferential (circum.) directions, respectively (i.e., Protocol 5 in Table 1) for the control group. Shaded regions represent standard error.
